# Effect of Simultaneous Bilingualism on Speech Intelligibility across Different Masker Types, Modalities, and Signal-to-Noise Ratios in School-Age Children

**DOI:** 10.1371/journal.pone.0168048

**Published:** 2016-12-09

**Authors:** Rachel Reetzke, Boji Pak-Wing Lam, Zilong Xie, Li Sheng, Bharath Chandrasekaran

**Affiliations:** 1 Department of Communication Sciences and Disorders, The University of Texas at Austin, Austin, Texas, United States of America; 2 Institute for Neuroscience, The University of Texas at Austin, Austin, Texas, United States of America; 3 Cognitive Neuroscience, The University of Texas at Austin, Austin, Texas, United States of America; Universitat Zurich, SWITZERLAND

## Abstract

Recognizing speech in adverse listening conditions is a significant cognitive, perceptual, and linguistic challenge, especially for children. Prior studies have yielded mixed results on the impact of bilingualism on speech perception in noise. Methodological variations across studies make it difficult to converge on a conclusion regarding the effect of bilingualism on speech-in-noise performance. Moreover, there is a dearth of speech-in-noise evidence for bilingual children who learn two languages simultaneously. The aim of the present study was to examine the extent to which various adverse listening conditions modulate differences in speech-in-noise performance between monolingual and simultaneous bilingual children. To that end, sentence recognition was assessed in twenty-four school-aged children (12 monolinguals; 12 simultaneous bilinguals, age of English acquisition ≤ 3 yrs.). We implemented a comprehensive speech-in-noise battery to examine recognition of English sentences across different modalities (audio-only, audiovisual), masker types (steady-state pink noise, two-talker babble), and a range of signal-to-noise ratios (SNRs; 0 to -16 dB). Results revealed no difference in performance between monolingual and simultaneous bilingual children across each combination of modality, masker, and SNR. Our findings suggest that when English age of acquisition and socioeconomic status is similar between groups, monolingual and bilingual children exhibit comparable speech-in-noise performance across a range of conditions analogous to everyday listening environments.

## Introduction

Speech communication rarely occurs in favorable listening conditions. Take, for example, the classroom. Various noise types, such as the loud heating and cooling system or students chatting in an adjacent hallway, compete with target speech signals and critically tax perceptual, linguistic, and cognitive processes. 20% of school-age children in the United States speak a language other than English at home, and this percentage is predicted to increase in the coming years [1]. Emerging work has shown that bilinguals demonstrate task-specific perceptual and cognitive advantages relative to monolinguals [2, 3], and disadvantages in some linguistic processes such as lexical retrieval and vocabulary knowledge (for a review see, [4]). Given the disparity between bilinguals and monolinguals on these fundamental processes, do the groups significantly differ in recognizing speech in challenging listening conditions? This question becomes further complex when considering bilingual children who are in the process of acquiring two languages at the same time [5]. Most of the existing literature on speech-in-noise abilities in bilingual children has focused on children for whom English is their second language [6, 7]. While these studies are important, there is a significant gap in speech-in-noise evidence for bilingual children who learn two languages *simultaneously*. The goal of the present study was to investigate the extent to which simultaneous bilingualism in school-age children impacts English sentence recognition in noise using a comprehensive battery that tests sentence recognition under various adverse listening conditions that differentially affect perceptual and cognitive processes.

### Speech perception in noise loads on perceptual, cognitive, and linguistic processes

Successful communication in challenging listening conditions requires a complex interdependence among perceptual, cognitive, and linguistic processes. In order to select a desired signal from competing sound sources, the auditory system must extract key acoustic features from the incoming stimulus stream. Interference with perceptual processes occurs when degradation caused by background noise renders portions of the target signal imperceptible [8]. In turn, this may lead to challenges in mapping key acoustic features to phonetic representations, and consequently to lexical representations (for a review see, [9]). A large body of work also implicates a role for cognitive processes in the extraction of speech from background noise. For example, the ease of language understanding (ELU) model indicates that for speech comprehension to occur sublexical information is Rapidly, Automatically, and Multimodally Bound into a PHOnological representation (RAMBPHO), a type of temporary storage system [10]. Poor speech signal quality due to noise results in ambiguous information in RAMBPHO which cannot be easily matched to stored speech representations. In such cases, working memory is needed to explicitly reprocess the incoming speech signal to resolve the mismatch. Working memory, a subcomponent of executive function [11], is engaged during speech recognition in adverse listening conditions to temporarily store salient acoustic cues, such as the fundamental frequency of the target speaker [9]. Cognitive demands tend to be greatest when the competing sound source has meaningful content, e.g. informational maskers [9, 12–14]. Like energetic masking (e.g. steady-state noise), informational masking involves selective attention and signal separation in the auditory periphery. However, recognizing speech in informational maskers not only requires listeners to cope with signal degradation due to energetic masking, but also to expend greater cognitive resources to ignore the meaningful distractors and attend to the target signal [13, 15]. Finally, speech recognition in challenging listening conditions is also dependent upon linguistic factors, such as vocabulary knowledge and lexical access. For example, a recent study demonstrated a positive correlation between phonemic restoration benefit and receptive vocabulary size and verbal intelligence, as measured by the Peabody Picture Vocabulary Test (PPVT-III-NL[16])[17]. The ELU additionally posits that individual differences in the quality of speaker-internal phonological representations in long-term memory also contributes to individual differences in the perception of speech in adverse listening conditions [10]. Taken together, these findings suggest that linguistic knowledge significantly contributes to the restoration of degraded speech.

### Bilingualism differentially impacts perceptual, cognitive, and linguistic processes

The bilingual experience differentially shapes perceptual, cognitive, and linguistic mechanisms across the lifespan resulting in outcomes ranging from better to poorer performance, when compared to monolingual age-matched peers [4]. Constant communication in two languages can fine-tune subcortical auditory responses to incoming speech signals [18]. Bilinguals have also shown advantages in executive function (specifically, selective attention and inhibitory control) across the lifespan [19–23], with evidence suggesting that these processes emerge earlier in bilingual children, compared to age-matched monolingual peers [21]. These studies have shown that bilingual children are less distracted by irrelevant stimulus features compared to monolinguals, and in turn demonstrate faster or more accurate identification of target stimulus features [20, 24]. Finally, studies investigating verbal fluency and lexical retrieval often report poorer performance in bilinguals relative to monolingual peers [25–28]. The exact reason for the corroborated bilingual deficit in verbal fluency and lexical access is unclear. Some studies have suggested that verbal fluency differences are ameliorated between monolinguals and bilinguals when language proficiency is matched between groups [29]. Other studies that have specifically investigated lexical access abilities in bilinguals and monolinguals suggest that poorer performance in bilinguals may arise from greater linguistic processing demands due to competition between two lexicons [30]. This assumption stems from a large body of evidence that indicates that bilinguals co-activate both languages during language comprehension [30, 31].

As reviewed, bilingualism differentially impacts perceptual, cognitive, and linguistic processes. Therefore, it can be argued that a significant advantage or disadvantage in one or more of these underlying perceptual, cognitive (e.g. better executive control), or linguistic processes (e.g. poorer vocabulary knowledge), may contribute to differences in speech perception in noise performance for bilingual listeners compared to monolingual peers. Likewise, performance differences between groups may arise across varied speech-in-noise experimental designs, since speech perception in noise loads on perceptual, cognitive, and linguistic processes in distinct ways through different listening conditions (e.g. various noise levels and noise types).

### Bilingualism and audio-only speech perception in noise: Mixed evidence

Prior work has revealed mixed evidence for the impact of bilingualism on audio-only speech-in-noise performance. Some studies have shown that early and simultaneous bilingual listeners exhibit poorer performance on speech-in-noise tasks relative to age-matched monolingual peers [32–34]. For example, one study investigated the impact of early bilingualism on speech perception in noise. While there was no difference between Spanish-English bilingual (age of English acquisition: ≤ six yrs.) and monolingual participants’ monosyllabic word perception in quiet, bilingual participants exhibited lower performance than monolingual participants in noise conditions. In another study, the Speech Perception in Noise (SPIN) test [35] was used to investigate the impact of speech noise and reverberation on the ability to recognize high- and low-predictable words in five groups of adult listeners who differed in their age of English acquisition [33]. The results revealed that while simultaneous and early bilinguals (age of English acquisition ≤ 5–7 yrs.) performed comparably to monolingual controls in mildly degraded listening conditions, monolinguals outperformed the two groups in more challenging listening conditions. In sum, these findings suggest that differences between monolingual and bilingual performance on speech-in-noise tasks may only be observable in conditions with high task demand (i.e. more challenging listening conditions). The results also indicate that age of English acquisition is a significant predictor of bilingual listeners’ identification of degraded target words in various combinations of noise, reverberation [33, 34] and context [33].

In contrast, recent evidence has demonstrated that simultaneous bilinguals exhibit *comparable* performance on speech-in-noise tasks, relative to monolingual age-matched peers [36, 37]. For example, one study found similar performance between English monolingual and simultaneous Spanish-English children (age of English acquisition ≤ 5 yrs.) on a forced-choice, picture-pointing paradigm that assessed speech reception thresholds across both English and Spanish two-talker masking conditions, as well as spectrally matched noise [37]. In another study with young adult listeners similar performance was found between English monolingual and simultaneous Greek–English bilingual listeners (age of English acquisition ≤ 2 yrs.) for recognition of target words across both English and Greek speech masker types [36]. In contrast to studies that revealed poorer speech-in-noise performance for bilinguals [33, 34], these studies [36, 37] suggest that simultaneous bilingualism does not negatively impact speech perception in noise. Several methodological variations can be noted in the type of target stimuli selected (e.g. monosyllabic words, SPIN sentences, BEL sentences), the maskers implemented (e.g. reverberation, spectral noise, various multi-talker babble tracks), as well as the SNR at which the stimuli were presented (e.g. +4 dB to -5 dB SNR).

These conflicting results and methodological variations pose a challenge to clinicians who need to determine the extent to which English speech-in-noise assessment tools can reliably test bilingual listeners with early English language acquisition [38]. Further, with the exception of a single study [37], there is a dearth of evidence for simultaneous bilingual children’s performance on English speech recognition in noise tasks.

### Bilingualism and audiovisual speech perception in noise: lack of evidence

The existing studies on early and simultaneous bilingual speech-in-noise performance have only considered the contribution of auditory information to speech perception in adverse listening conditions. This is surprising since it is well-established that viewing a speaker’s articulatory movements improves speech-in-noise for both monolingual and non-native listeners [39–41]. Currently, the role of visual cues in bilingual speech perception has been examined in infants simultaneously learning two languages [42–44] and early bilingual adult listeners perceiving speech in a non-native language [41, 45–50]. In sum, these studies reveal that although bilingual infants show an audiovisual *advantage* relative to age-matched monolingual counterparts, bilingual adults do not. For example, bilingual infants exhibit better discriminability of silent talking faces [42, 44], and also exploit audiovisual speech cues more than monolingual infants [43]. These observations have led some to propose that bilingual infants may capitalize on audiovisual cues more than monolinguals to disambiguate their native languages as they simultaneously acquire two languages [51]. In contrast, studies that have investigated audiovisual phoneme identification in bilingual adults have shown that although monolingual and bilingual listeners both benefit from visual cues, monolingual adults outperform bilingual age-matched peers [50]. The evidence here suggests that reliance on audiovisual cues may change as a function of age in bilinguals. The use of audiovisual speech cues should be investigated in *school-age children* to gain a better understanding of this developmental change in reliance on visual cues in bilinguals since evidence is current lacking for this age group.

### Bilingualism and audiovisual speech perception in noise: Study design and motivation

As reviewed, prior studies have yielded mixed results regarding bilingual speech perception in noise performance across different experimental designs. While this body of evidence is important, these studies have not examined audiovisual speech recognition in noise for bilingual listeners. Expanding on previous research, we investigated the effect of simultaneous bilingualism through a comprehensive speech-in-noise battery that assessed sentence recognition across: different modalities [audio-only (AO), audiovisual (AV)], since monolingual and bilingual listeners have been found to rely on visual cues differently across the lifespan; and two masker types that distinctively engage cognitive processes (energetic masker: steady-state pink noise; informational masker: two-talker babble). Perceiving speech in competing information maskers is more critically dependent upon executive processes, such as selective attention and inhibitory control [9], cognitive processes for which bilingual children have demonstrated advantages [20, 23, 52]. Finally, our comprehensive speech-in-noise battery assessed sentence recognition across a more expanded range of signal-to-noise ratios (SNRs; 0 to -16 dB) in school-age children. The implementation of more challenging SNRs has been found to tax the auditory system in such a way to elicit individual differences in the breakdown of speech sound encoding that may not otherwise occur in more favorable listening conditions [53, 54]. To control for linguistic bias between groups, we recruited a group of simultaneous bilingual children with similar linguistic backgrounds as similultaneous bilingual participants reported in [37] for: English language onset (age of English acquisition ≤3 years), as well as proficiency and usage of both languages (see Table 1). We also ensured that our two groups were matched on socioeconomic status (SES), since SES and bilingualism are found to contribute independently to cognitive and linguistic development [23, 55]. Prior studies have not matched monolingual and bilingual groups on SES [6, 7, 33, 34, 37]. Finally, we utilized the BEL sentences, which were developed to have simpler English lexicon and syntax compared to other standardized speech-in-noise sentences [56]. With this experimental design, we aimed to assess whether performance differences between monolingual and simultaneous bilingual school-age children, if present, are restricted to certain listening conditions when socioeconomic status and age of English acquisition is similar between groups.

**Table 1:** Age of English acquisition (AoEA), daily language usage, and reported language proficiency of the twelve bilingual participants.

				% of hours of use		Speaking		Comprehension	
				(per day)		Proficiency		Proficiency	
Participant	Age at testing (years)	Sex	AoEA (months)	Other Language	English	Other Language	English	Other Language	English
1	6	Female	18	25	75	4	5	5	5
2	7	Female	12	30	70	5	5	4	5
3	6	Male	20	30	70	5	5	5	5
4	7	Male	36	35	65	5	5	4	4
5	7	Male	36	20	80	5	5	5	5
6	8	Female	0	25	75	4	5	4	5
7	10	Female	0	25	75	4	5	4	5
8	6	Male	0	30	70	5	5	5	5
9	7	Female	0	25	75	3	5	4	5
10	8	Female	0	25	75	4	5	4	5
11	9	Female	36	40	60	4	4	4	4
12	7	Female	36	40	60	4	4	4	4

*Note*. Parents reported the estimated percentage of hours per day that both languages were used within the past 24 months. Speaking Proficiency: parents reported the extent to which their child could be understood in English and the other language on the following scale: 1 = *does not speak any;* 2 = *a couple of words or phrases*; 3 = *can have a simple conversation;* 4 = *frequently;* 5 = *all of the time*. Comprehension Proficiency: parents reported how often their child could understand English and the other language on the following scale: 1 = *does not understand anything*; 2 = *understands a couple of words or phrases*; 3 = *understands basic directions*; 4 = *understands the majority of what they are told*; 5 = *understands everything they are told*.

## Materials and Methods

All materials and procedures were approved by the Institutional Review Board at the University of Texas at Austin. All children, as well as their parents, provided written informed consent before their participation in the study.

### Participants

Twenty-four elementary school-age children (age range: 6–10 years) from the Austin community participated in the experiment. Inclusionary criteria consisted of: normal hearing defined as hearing thresholds < 20 dB, normal hearing level (nHL) for octaves from 250 to 8000 Hz, no history or current diagnosis of a speech, language, or neurodevelopmental disorder (confirmed via parent report), and normal intelligence (*M* = 100, *SD* = 15), as measured by the Kaufman Brief Intelligence Test-Second Edition, KBIT-2, matrices subtest [57]; monolinguals: *M* = 104.83, *SD* = 17.02; bilinguals: *M* = 118.42, *SD* = 19.51; [*F*(1,22) = 3.30, *P* = 0.08]. The KBIT-2 matrices subtest assesses non-verbal intelligence through the evaluation of an individual’s ability to perceive relationships and complete visual analogies [57]. Monolinguals (n = 12; 6 female) and simultaneous bilinguals (n = 12; 8 female) were matched on age (monolinguals: *M* = 7.33 yrs, *SD* = 1.23 yrs; bilinguals: *M* = 7.33 yrs, *SD* = 1.23 yrs; [*F*(1,22) = 0, *P* = 1]) and family socioeconomic status (Family-SES; monolinguals: *M* = 55.75, *SD* = 6.18; bilinguals: *M* = 54.77, *SD* = 14.36; [*F*(1,22) = 0.05, *P* = 0.83]). The Family-SES score was calculated based on the Four Factor Index of Social Status [58] This metric takes into account parents’ occupation, education, sex and marital/cohabitation status and returns a six-tier classification of social strata based on scores ranging from 8 to 66. All participants’ family social strata fell into two categories: (a) medium business, minor professional, technical (social stratum range = 40–54), or (b) major business and professional (social stratum range = 55–66). SES is an important factor to control, as SES and bilingualism have both been found to contribute independently to cognitive and linguistic development [23].

Each parent completed a language history questionnaire [59]. Results derived from this language history questionnaire have been found to correlate well with linguistic proficiency [37]. Through the questionnaire parents rated their child’s speaking and comprehension proficiency on a scale ranging from 1 to 5 (see Table 1). The questionnaire specified that speaking proficiency referred to how easily the child could be understood in each language, while comprehension proficiency indicated how easily the child could understand each language. The 12 bilingual speakers consisted of 7 Chinese-English, 4 Arabic-English, and 1 English-Spanish participant. All parents of bilingual participants reported that their child was born in the United States, except one participant. The parent of this participant reported that the child was born in Japan, and soon after, the family immigrated to the United States. Five participants were reported to start acquiring both languages from birth, three before 20 months, and four before 36 months of age. All parents reported that their child’s use of the other language exceeded 20% throughout the day (see Table 1 for detailed demographics of bilingual participants).

Based on previously reported bilingualism classification, we labeled these children as simultaneous bilinguals. The parents of the 12 monolingual speakers stated that their child began acquiring English from birth and was not exposed to a second language throughout the child's lifespan.

### Target Stimuli

Disadvantages for bilinguals, relative to monolinguals, have been found for vocabulary knowledge (for reviews, [4, 22]). Therefore, it is unclear if poor speech-in-noise performance in bilinguals arises because of poor linguistic proficiency in the target language. To control for the influence of linguistic knowledge on speech perception in noise, we selected 80 target sentence stimuli from the Basic English Lexicon [56]. The BEL corpus was developed for native and non-native English speech-recognition testing with the specific aim to select lexical items that would be familiar to non-native listeners who may have limited knowledge of English lexicon and syntax [56]. This complete corpus consists of 20 lists of 25 sentences. The 80 sentences that were selected for the current experiment comprised of simple vocabulary that would be present in an elementary school-age child’s lexicon (e.g. “mouse”, “cheese”, “rabbit”, “toy”, “tree”). Each sentence contained four keywords for intelligibility scoring. For example, “*The* ***black***_***1***_ ***cat***_***2***_ ***climbed***_***3***_ *the* ***tree***_***4***_.” A target word was counted incorrect if the child added or deleted morphemes. For example, if the child reported: "cats" for "cat” or “climb” for “climbed," the target words would be scored as incorrect. One male native speaker of American English was recorded producing the full set of 80 sentences. The audio was recorded at a sampling rate of 48000 Hz. The video and audio for each sentence were segmented and separated, and the audio tracks were equalized for RMS amplitude using Praat [60]. Similar stimuli have been utilized across a range of studies from our lab (e.g. [41, 61, 62]).

### Maskers

The protocol for creating the maskers, as well as mixing the target speech with maskers, was additionally consistent with methodology reported in these past publications. There were two masker types created for this experiment: two-talker babble and steady-state pink noise. For the two-talker babble tracks, two male native speakers of American English were recorded in a sound-attenuated booth at Northwestern University as part of the Wildcat Corpus project [63]. Each speaker produced a set of 30 simple, meaningful English sentences (from [64]). All sentences were segmented from the recorded files and equalized for RMS amplitude in Praat [60]. The sentences from each talker were concatenated in random order to create 30-sentence strings without silence between the sentences. Two of these strings were mixed using the mix paste function in Audacity (Version 1.2.5; www.audacity.sourceforge.net) to generate two-talker babble. A second masker track consisting of 10 seconds of steady-state pink noise was created using the Noise Generator option in Audacity 1.2.5. Both masker tracks were equated for RMS amplitude to 50dB, 54dB, 58dB, 62dB and 66dB to create ten discrete, long masker tracks. Each masker track was segmented using Praat to create 80 unique noise clips, for a total of 5 noise clips per target sentence per masker type.

### Mixing targets and maskers

Each audio clip was mixed with the five corresponding noise clips from each masker track using Adobe Audition to create ten final stimuli of the same target sentence with the following signal to noise ratios: 0 dB, -4 dB, -8 dB, -12 dB, and -16 dB. The mixed audio clips served as the stimuli for the audio-only condition. For the audiovisual condition, audio clips were reattached to the corresponding videos using Final Cut Pro. A freeze frame of the speaker was displayed during the 500 ms noise leader and 500 ms noise trailer. The final mixed target sentence with masker was approximately 2000 ms. In total, for each noise type, there were 400 final audio files (80 sentences × 5 SNRs) and 400 corresponding audiovisual files (80 sentences × 5 SNRs).

### Procedures

#### Speech perception in noise

Participants completed the experiment in a sound attenuated booth. The sentence stimuli were binaurally presented to participants through Sennheiser HD-280 Pro headphones. A trained research assistant was present to type the verbal responses of each child and to ensure that the child attended to the computer screen through the entire duration of the experiment. Four sentences with 16 keywords for scoring were presented in each combination of masker (stead-state pink noise, two-talker babble), SNR (0 to -16 dB), and presentation modality (AO, AV). The presentation order was randomized. The child was instructed first to listen to the sentence produced by the speaker and then repeat the exact sentence that they heard out loud. The research assistant further informed the child that the speaker would begin talking after the noise started. Finally, the child was instructed that even if they only heard a few words, to say those words out loud, and if they were unsure of what they heard, to make their best guess. If they did not understand any words, they were asked to say ‘X.' The speech perception in noise task lasted approximately 15–20 minutes, and the administration of the Kaufman Brief Intelligence Test-Second Edition (KBIT-2) [57] also lasted approximately 15–20 minutes. In sum, the total experimental session lasted approximately 35–40 minutes.

#### Data Analysis

The speech intelligibility data was analyzed with logistic mixed effects modeling implemented in lme4 package using a binomial logit link in R [65]. This type of analysis models mixed effects logistic regression, where the estimates of the model output correspond to the log odd or probability of producing a correct response. We have utilized mixed effects modeling in past speech-in-noise publications from our laboratory [41, 61, 66]. In the current analyses, participants' target word identification in sentences was coded as "correct" or "incorrect" on each trial. This trial-by-trial accuracy was treated as the dichotomous dependent variable. Differences in child speech-in-noise performance have been reported for speech masked by competing talkers compared to speech masked by steady-state noise [12, 67–69]. To confirm this result, a simple linear regression analysis was conducted to examine differences in the proportion of correct keywords identified between the two masker types in the current study. The analysis indicated a significant effect of masker type, *F* (1,46) = 12.84, *P* = <0.001, *R*^*2*^ = 0.22, *b* = 0.04, suggesting that the proportion of correct keyword identification was higher in the steady-state, pink noise masker condition compared to the two-talker maker condition. Therefore, we conducted two separate mixed effects analyses to investigate language group differences in the two-talker and steady-state pink noise masker conditions. The fixed effects of interest were SNR, modality [AO (reference level) versus AV], and listener group [monolingual (reference level) versus bilingual], and their interactions. SNR was mean-centered and treated as a continuous variable. Modality and listener group were treated as categorical variables. In both models, subjects and sentences were specified as random factors to account for individual differences and potential linguistic variation among sentences, respectively. Two alternative random effects structures were considered to determine the optimal model: (1) by-sentence intercept and by-subject sentence slope, and (2) by-subject and by-sentence intercepts [41]. The first model failed to converge, therefore the second model was used.

## Results

**Fig 1:**
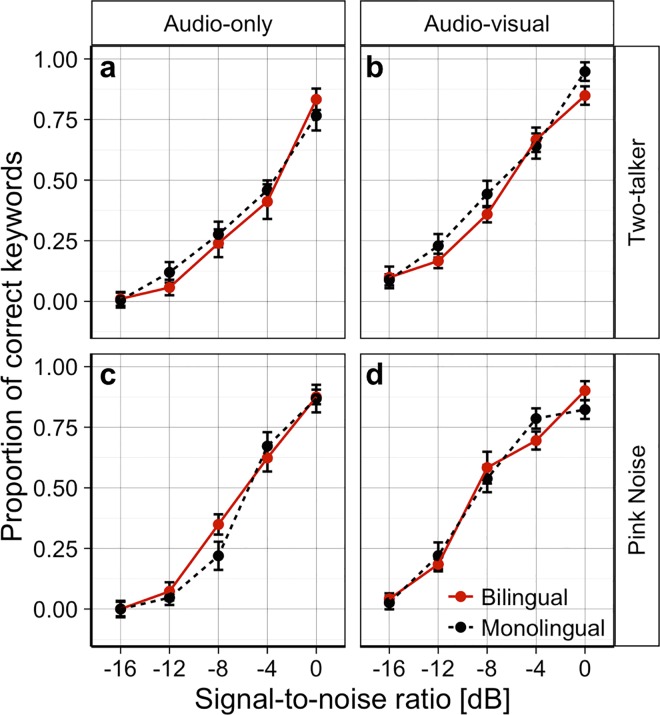
Mean proportion of correct keywords as a function of signal-to-noise ratio across all conditions. The top panels show the mean proportion of correct keywords identified by bilingual (red) and monolingual (black dashed) school-age children in the two-talker masker audio-only (a) and audio-visual (b) conditions. The bottom panels show the mean proportion of correct keywords identified by the two groups in the steady-state pink noise masker audio-only (c) and audio-visual (d) conditions. Error bars denote one standard error from the mean.

Fig 1 shows the mean proportion of correctly identified target words as a function of SNR in monolingual and bilingual children across the four masker-modality conditions.

### Steady-state pink noise masker

In the mixed-effects model, the intercept was significant, *b* = -3.65, *SE* = 0.36, *Z* = -10.02, *P* < 0.001. The simple effect of language group was not significant, *b* = 0.60, *SE* = 0.42, *Z* = 1.42, *P* = 0.156, indicating that proportion of correctly identified target words in noise were not significantly different between the two language groups. The simple effect of SNR was significant *b* = 0.53, *SE* = 0.03, *Z* = 17.02, *P* < 0.0001, where improving SNR increased the probability of correct target word identification. The simple effect of modality was significant, *b* = 1.79, *SE* = 0.26, *Z* = 6.87, *P* < 0.0001, suggesting that the probability of correct target word identification was significantly higher in AV compared to the AO condition. The SNR by modality interaction was significant, *b* = -0.12, *SE* = 0.04, *Z* = -3.28, *P* = 0.001, indicating that slope of increase for intelligibility benefit from the improvement of SNR was less for the AV condition compared to the AO condition. There were no other significant two- or three-way interactions.

### Two-talker masker

In the mixed-effects model, the intercept was significant, *b* = -3.37, *SE* = 0.44, *Z* = -7.65, *P* < 0.0001. The simple effect of language group was not significant, *b* = -0.46, *SE* = 0.56, *Z* = -0.82, *P* = 0.414, demonstrating that proportion of correctly identified target words in noise was not significantly different between the two language groups. The simple effect of SNR was significant, *b* = 0.42, *SE* = 0.03, *Z* = 16.10, *P* < 0.0001, where improving SNR increased the probability of correct target word identification. The simple effect of modality was significant, *b* = 1.34, *SE* = 0.24, *Z* = 5.57, *P* < 0.0001, suggesting that target words in sentences were more correctly identified in AV relative to AO conditions. There were no significant two- or three-way interactions.

## Discussion

Prior studies have found mixed evidence for the impact of bilingualism on speech-in-noise performance. One body of literature has demonstrated that early and simultaneous bilingual listeners demonstrate poorer performance on speech-in-noise tasks relative to age-matched monolingual peers [33, 34]. In contrast, recent studies have found no difference in speech-in-noise performance between monolingual and simultaneous bilingual listeners [36, 37]. Here we examined the extent to which speech-in-noise performance differences, if present, were modulated by certain listening conditions in monolingual and simultaneous bilingual children. To that end, we assessed the impact of simultaneous bilingualism on speech perception through a comprehensive speech-in-noise battery, across different modalities, masker types, and SNRs. Our results revealed no difference in performance between monolingual and simultaneous bilingual children (age of English acquisition ≤ 3) in each combination of presentation modality (AO, AV), masker (steady-state pink noise, two-talker babble), and SNR (0 to -16 dB SNR).

The main effects of the tested factors observed in the current study are in line with previous studies examining speech intelligibility in adverse listening conditions in samples of school-age children. The two-talker masker condition was more difficult for all children, compared to the steady-state pink noise condition [12, 67–69]; all listeners exhibited better keyword identification in sentences masked by easier SNRs [70–73], and AV speech was more intelligible than AO speech [39, 40, 50, 74].

Apart from typical developmental factors and noise, research has demonstrated that the language experience of children further affects their ability to recognize speech-in-noise [7]. Successful recognition of the speech signal from speaker to listener has been described in the literature through signal-dependent and signal-independent factors [9]. Signal-dependent factors are related to the quality of the incoming speech signal (e.g. the level and type of background noise). In contrast, signal-independent factors refer to characteristics internal to the listener (e.g. the linguistic proficiency of the listener or familiarity with the content being conveyed by the speaker) [9, 75]. In the current study, our findings indicate that when signal-independent factors are similar between monolinguals and bilinguals, such as comparable early exposure to the target language and socioeconomic status, both groups exhibit the same degree of speech-in-noise difficulties across a range of signal-dependent factors (i.e. different noise types and noise levels). SES is another important signal-independent factor that was matched between groups in the current study. The fact that SES was similar between groups may be another reason the current study did not yield poorer speech-in-noise performance for bilingual participants; a finding that was previously shown in speech-in-noise studies that did not control for SES [6, 7, 33, 34]. This assumption is supported by recent evidence that performance on linguistic and cognitive tasks is uniquely influenced by SES and bilingualism [23].

The current results are in contrast to prior studies that have demonstrated poorer audio-only speech-in-noise performance in early and simultaneous bilingual performance relative to monolingual peers [32–34]. The discrepancy in findings across studies may be due to differences in target stimuli used. Studies that have shown lower speech-in-noise performance in early and simultaneous bilinguals have utilized different English monosyllabic word and sentence stimuli [32–34] from studies that did not demonstrate differences in speech-in-noise performance between groups [36, 37]. For example, some studies [32, 33] implemented English sentences from the Speech-Perception-in-Noise (SPIN) test [35, 76], while others [37] employed target sentences from the Basic English Lexicon (BEL) corpus. Like [37], the present study implemented target BEL sentences across different masking conditions and did not find differences between monolingual and simultaneous bilingual school-age children. The findings from the current study suggest that the simpler lexicon and grammar in BEL sentences may eliminate linguistic bias in the testing of bilingual children simultaneously learning two languages, compared to other available speech-in-noise test materials. However, one limitation of the present study is that there was no direct comparison between the perception of BEL sentences and the perception of sentences from another standardized speech-in-noise measure. Future studies should empirically compare speech-in-noise abilities in simultaneous bilingual children perceiving BEL sentences in a comprehensive speech-in-noise battery, relative to the perception of other clinically implemented English speech-in-noise sentences.

The cross-study differences in early and simultaneous bilingual speech-in-noise performance may also be due to differences in English language learning environments across bilingual participant samples. For example, the majority of the simultaneous bilingual children in the current study, like the majority of participants in studies that also did not show bilingual disadvantages for speech-in-noise [36, 37], were born and raised in the United States. The majority of other bilingual speech-in-noise studies did not report the country of birth of their participants [33, 34]. Therefore, it is unclear whether the bilingual participants who took part in these studies were born and raised in the United States, or whether they emigrated from another country. If the subjects emigrated from another country, even at an early age, this would have an impact on the English language development of the participants. This is because it is well-established that the amount of language input strongly affects the rate of language growth for both monolingual and bilingual children [55, 77, 78].

Our results across different masker types in the AO conditions are in line with recent work that has demonstrated no difference in speech recognition performance for simultaneous bilinguals compared to age-matched monolingual peers across both speech (i.e. two-talker babble) and steady-state noise [36, 37]. Early bilinguals’ lower performance on speech perception in steady-state noise has been explained as the outcome of increased linguistic processing demand [34]. These linguistic demands have been suggested to arise from competition between two co-activated lexicons, and in turn, lead to difficulties in sound-to-meaning mapping in challenging listening environments. Such increased linguistic demands would also predict poorer speech perception in speech noise bilingual performance, relative to monolingual peers. On the other hand, bilingual advantages in cognitive processes, such as selective attention and inhibitory control, should lead to better performance for bilingual speech-in-noise–especially, in perceiving speech in competing talkers. Speech perception in speech noise critically depends on ignoring irrelevant information while selecting target information, cognitive abilities that have been found to be enhanced in bilingual children [20, 24]. This hypothesis is further supported by the ease of language understanding model (ELU) [10], which posits that individual differences in the quality of speaker-internal working memory capacity contributes to individual differences in the recovery of degraded acoustic information in noise. [10].

The current findings did not reveal either of the previously observed differences between language groups, even at more difficult SNRs. Similar bilingual and monolingual performance demonstrated in the two-talker babble conditions in the current study may be evidence for an optimum point between increased linguistic demands, and enhanced executive function in simultaneous bilinguals. This finding may also suggest that the bilingual advantage in executive function, which has been mostly demonstrated through non-linguistic tasks [20], does not generalize to the specific task demands of perceiving speech in noise. This idea is in line with a recent review which concluded that managing two languages does not result in general executive function advantages. The review instead suggests that a bilingual advantage in executive function may not exist at all or may be confined to very specific task-dependent circumstances [79]. Although the current experimental design presented sentences in both steady-state noise and two-talker babble across a range of more challenging SNRs compared to those implemented in past studies, it is likely that this task was still not sensitive enough to elicit either of the previously observed differences between language groups. For example, prior studies that have demonstrated poorer performance in early and simultaneous bilinguals due to other environmental acoustic factors (e.g. reverberation). Therefore, additional research is needed to better understand the extent to which other aspects of speech in noise processing modulate differences between monolingual and simultaneous bilingual listeners.

To the best of our knowledge, the current study is the first to evaluate the contribution of visual cues during speech perception in noise in school-age simultaneous bilingual children. We assessed the extent to which simultaneous bilingual children would use visual cues differently during speech-in-noise tasks, compared to age-matched monolingual peers. Our results demonstrated that simultaneous bilingual and monolingual children benefited equally from visual cues during speech perception in noise. While bilingual infants have been found to exploit audiovisual speech cues [43], bilingual adults have exhibited less reliance on audiovisual phoneme discrimination tasks, compared to monolingual peers [50]. In considering the current evidence with these prior findings, it may be suggested that as bilinguals develop from infancy to early childhood and become proficient in both languages, the need to capitalize on audiovisual cues to discriminate their two languages becomes less necessary [80].

Similar to communicating across two languages, experience playing a musical instrument has also been found to lead to enhanced perceptual and cognitive abilities [81–83]. For example, enhanced neural responses to pitch changes during speech processing has been demonstrated for musicians relative to nonmusicians [84, 85]. These observed enhancements in musicians have been found to be contingent upon the extent of musical expertise and training [84, 86], just as observed bilingual benefits are dependent upon the degree of proficiency and usage of both languages. Like bilingualism, there is mixed evidence for the transfer of enhanced perceptual and cognitive skills, resulting from music training, to enhanced speech-in-noise [87–91]. Speech-in-noise benefit for musicians has been suggested to be task-dependent, with musicianship advantages found to emerge more in informational masking conditions relative to energetic masking conditions [87, 92], but not always [88]. The fact that different types and levels of auditory experiences result in different levels of speech-in-noise outcomes highlights the need for more carefully defined participant groups, and implementation of comprehensive speech-in-noise test batteries, with a range of masker types, modalities, and SNRs. These methodological considerations will allow for a better understanding of the interactions between varying degrees and types of auditory expertise (signal-independent factors) and speech perception in noise.

While the current study provides new insights into simultaneous bilingual performance on speech-in-noise, a few limitations remain. First, while a large body of evidence has shown that bilinguals demonstrate advantages in executive function (specifically, selective attention and inhibitory control) [19–23], we did not implement any cognitive measure to confirm these findings and relate them to speech-in-noise performance. Future work should investigate the links between bilingualism in school-age children and measures of executive function to provide better insight into how these cognitive enhancements, if present, relate to individual differences in speech perception in noise. Second, while we carefully controlled for differences in age, socioeconomic status, and non-verbal intelligence between the monolingual and bilingual groups, we did not investigate language-specific bilingual influences on speech-in-noise performance. Future studies should explore the extent to which differences in English speech perception in noise occur as a function of first language background (e.g. tonal vs. non-tonal language). A third limitation is the generalizability of these findings to the classroom setting. In the current study, we did not observe differences between monolingual and simultaneous bilingual children’s speech perception in noise across a range of listening conditions, using high-frequency target stimuli from the BEL corpus. The current study lays the foundation for future work to pursue more classroom oriented investigations, such as the extent to which monolinguals and bilinguals differ on novel word learning in classroom noise.

### Conclusion

In conclusion, our results indicate that when the age of English acquisition and socioeconomic status are similar between groups, monolingual and simultaneous bilingual children exhibit the same degree of speech-in-noise difficulties across a range of adverse listening conditions. For these simultaneous bilingual listeners, bilingualism does not negatively affect English speech recognition across a combination of masker (steady-state pink noise, two-talker babble), SNR (0 to -16 dB), and presentation modality (AO, AV). These findings suggest that despite increasing linguistic demands that may arise from two competing lexicons, simultaneous bilingual listeners have the ability to perform equally to monolinguals on the identification of degraded target words in masked sentences.
